# Supplementary material for: Serum BDNF levels and the antidepressant effects of electroconvulsive therapy with ketamine anaesthesia: a preliminary study
Source: PeerJ. 2021 Feb 5;9:e10699. doi: 10.7717/peerj.10699 (PMC7869666; doi:10.7717/peerj.10699)
Supplement: Supplemental Information 2 [file peerj-09-10699-s002.doc]

**Supplemental Table 1.** Correlation analysis of serum BDNF levels with HAMD-17 scores and BPRS scores

|  | Baseline HAMD-17 scores | Baseline BPRS scores | HAMD-17 scores after the last ECT | BPRS scores after the last ECT | Changes in HAMD-17 scores | Changes in BPRS scores |
| --- | --- | --- | --- | --- | --- | --- |
| Baseline serum BDNF levels | *r=*0.071 | *r*=0.162 | - | - | - | - |
|  | *P*=0.710 | *P*=0.392 |  | - | - | - |
| Serum BDNF levels after the last ECT | - | - | *r*=-0.198 | *r*=0.058 | - | - |
|  | - | - | *P*=0.294 | *P*=0.762 | - | - |
| Changes in serum BDNF levels | - | - | - | - | *r*=0.052 | *r*=0.004 |
|  | - | - | - | - | *P=0*.787 | *P*=0.983 |
| Abbreviations: BDNF=brain-derived neurotrophic factor; BPRS=the Brief Psychiatric Rating Scale; ECT=electroconvulsive therapy; HAMD-17=the 17-item [Hamilton Depression Rating Scale](https://www.sciencedirect.com/topics/medicine-and-dentistry/hamilton-rating-scale-for-depression); r=Pearson coefficient of correlation. | | | | | | |

**Supplemental Figure 1.** Baseline serum BDNF levels in patients with TRD and healthy controls


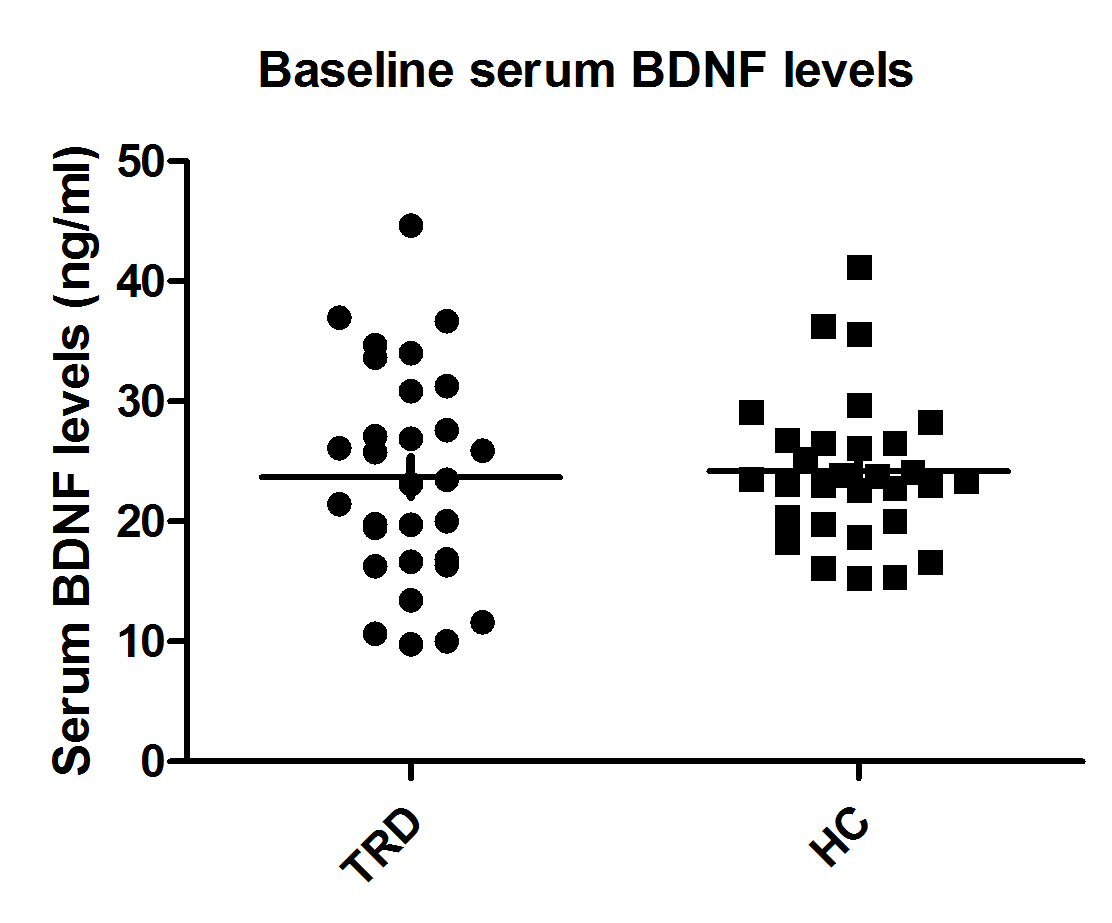


Notes: no significant difference was found between patients with TRD and healthy controls at baseline (*p>*0.05).

Abbreviations: BDNF=brain-derived neurotrophic factor; HCs=healthy controls; TRD=treatment-refractory depression.

**Supplemental Figure 2.** Changes in psychotic symptoms following eight ECT treatments


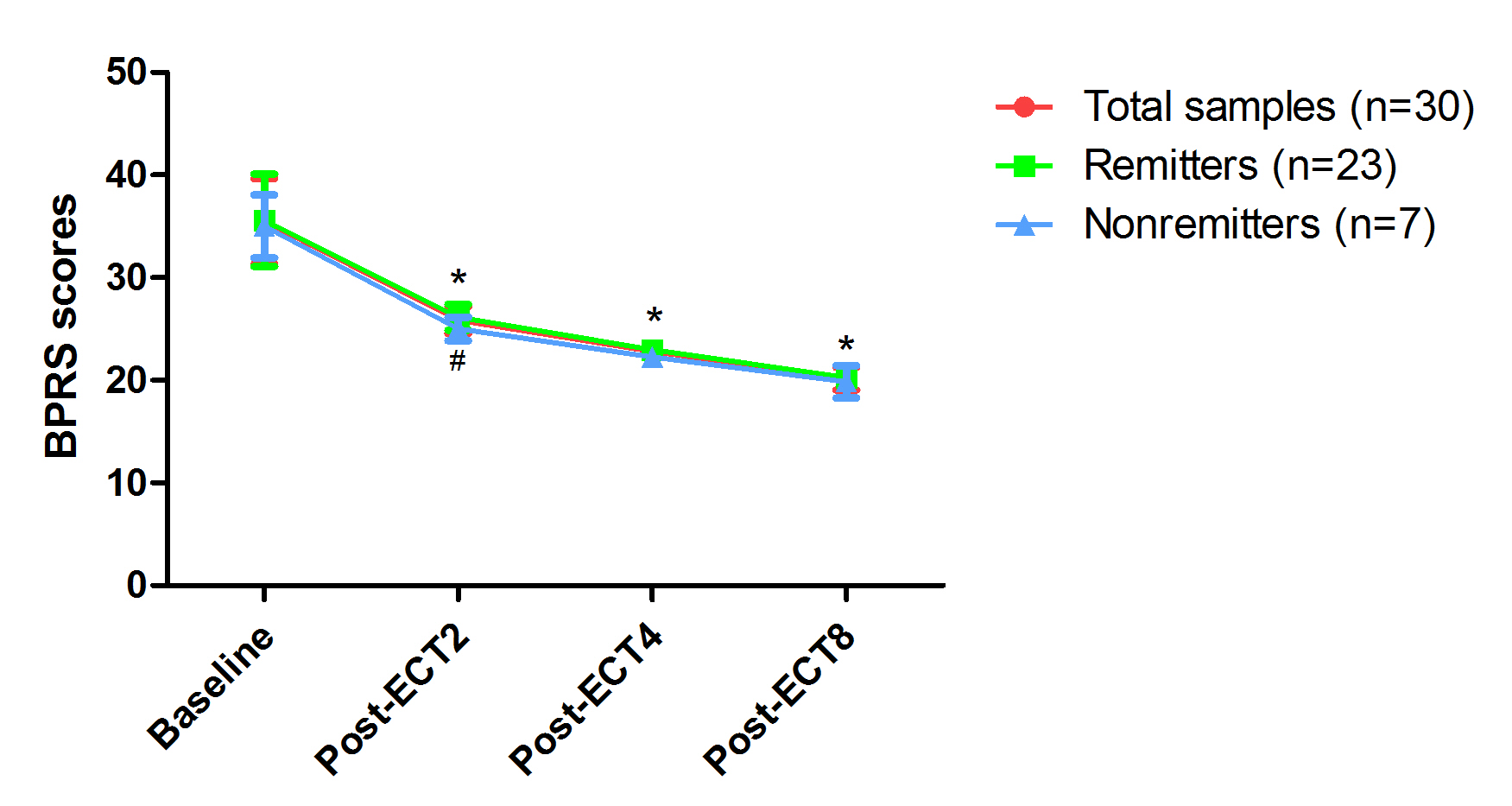


^*^Significant difference was found at indicated times when compared baseline (*p<*0.05).

^#^Significant difference was found between remitters and nonremitters at indicated times (*p<*0.05).

Abbreviations: ECT=electroconvulsive therapy; BPRS=the Brief Psychiatric Rating Scale.
